# PCSK9 Expression in Epicardial Adipose Tissue: Molecular Association with Local Tissue Inflammation

**DOI:** 10.1155/2020/1348913

**Published:** 2020-06-04

**Authors:** Elena Dozio, Massimiliano Ruscica, Elena Vianello, Chiara Macchi, Clementina Sitzia, Gerd Schmitz, Lorenza Tacchini, Massimiliano M. Corsi Romanelli

**Affiliations:** ^1^Department of Biomedical Sciences for Health, Università degli Studi di Milano, Via Mangiagalli 31, 20133 Milan, Italy; ^2^Department of Pharmacological and Biomolecular Sciences, Università degli Studi di Milano, Via Balzaretti 9, 20133 Milan, Italy; ^3^Institute for Clinical Chemistry and Laboratory Medicine, University of Regensburg, Franz-Josef-Strauß-Allee 11, 93052 Regensburg, Germany; ^4^Service of Laboratory Medicine1-Clinical Pathology, IRCCS Policlinico San Donato, Piazza E. Malan 1, 20097 San Donato Milanese, Milan, Italy

## Abstract

Epicardial adipose tissue (EAT) has the unique property to release mediators that nourish the heart in healthy conditions, an effect that becomes detrimental when volume expands and proinflammatory cytokines start to be produced. Proprotein convertase subtilisin/kexin type 9 (PCSK9), a proinflammatory mediator involved in atherosclerosis, is also produced by visceral fat. Due to the correlation of inflammation with PCSK9 and EAT enlargement, we evaluated whether PCSK9 was expressed in EAT and associated with EAT inflammation and volume. EAT samples were isolated during surgery. EAT thickness was measured by echocardiography. A microarray was used to explore EAT transcriptoma. The PCSK9 protein levels were measured by Western Blot in EAT and ELISA in plasma. PCSK9 was expressed at both the gene and protein levels in EAT. We found a positive association with EAT thickness and local proinflammatory mediators, in particular, chemokines for monocytes and lymphocytes. No association was found with the circulating PCSK9 level. The expression of PCSK9 in EAT argues that PCSK9 is part of the EAT secretome and EAT inflammation is associated with local PCSK9 expression, regardless of circulating PCSK9 levels. Whether reducing EAT inflammation or PCSK9 local levels may have beneficial effects on EAT metabolism and cardiovascular risk needs further investigations.

## 1. Introduction

Proprotein convertase subtilisin/kexin type 9 (PCSK9), one of the key regulators of the LDL receptor (LDLR), is a soluble member of the mammalian proprotein convertase family of secretory serine endoproteases [1]. While the liver is the main contributor to circulating PCSK9, other tissues produce PCSK9 pointing out its possible role beyond LDLR expression [2, 3]. PCSK9 seems to play a role in atherosclerotic plaque development, i.e., it is expressed in endothelial cells, regulates the migration ability of smooth muscle cells [4], and is raised by shear stress [5]. Circulating platelets have also been added to the list of PCSK9 targets [6], together with the description of a proinflammatory effect on macrophages [7].

It is well-established that body fat accumulation leads to metabolic diseases and increases cardiovascular (CV) risk [8]. Among different fat depots, the visceral epicardial adipose tissue (EAT) is an important risk factor for CV diseases (CVD), from atherosclerosis to cardiac remodeling, heart failure, valve calcification, and stenosis [9–16]. Although in physiological conditions, EAT nourishes the heart and plays protective functions, e.g., mechanical protection of coronaries, local energy store, and cryoprotection of the heart [17], when EAT volume expands, it becomes detrimental. These effects are mainly due to the release of bioactive molecules that affect the electrophysiological and contractile properties of cardiomyocytes and that promote cardiac remodeling, fibrosis, and coronary atherosclerosis progression [18]. Indeed, EAT lies in continuity to the myocardium and shares the same microcirculation. Vasocrine and paracrine interactions of bioactive molecules with coronary vessels and the myocardium exist. EAT hypertrophy promotes adipocyte degeneration and drives local inflammation through an increased infiltration of immune cells [19–21]. In particular, a rise in EAT adipocyte size has been associated with lipid droplet degeneration, the presence of macrophages polarized toward a proinflammatory M1 state, and the local expression of proinflammatory mediators [21]. Although EAT inflammation can increase, the levels of mediators produced locally may not correlate with the corresponding plasma levels [22].

EAT can be a proxy of overall visceral adiposity [23], and since this latter represents a potential source of PCSK9 [22], the present study is aimed at evaluating whether PCSK9 is expressed in EAT and if a correlation with EAT amount and local inflammation, regardless of total circulating levels, can be found. PCSK9 may therefore be ascribed among those bioactive molecules belonging to the EAT secretome affecting vascular homeostasis and heart health.

## 2. Materials and Methods

### 2.1. Study Population

A total of 68 patients with coronary artery disease (CAD) or valvular diseases (VR) and 46 control subjects matched for age, sex, and BMI (CTR) were enrolled in the study. Patients were recruited at the IRCCS Policlinico San Donato between October 2012 and July 2016. We followed the methods cited in our present paper by Dozio et al. [24]. We excluded patients with the following criteria: age ≤ 18 years, acute myocardial infarction in the previous month, end-stage heart failure, other heart diseases different from CAD and VR, malignant diseases, major abdominal surgery in the previous six months, renal and liver diseases, chronic inflammatory diseases, more than 3% change in body weight in the previous three months, and missing or incomplete clinical history and data. Among the 68 patients, 32 underwent open heart surgery. Twenty-one required coronary artery bypass surgery (CABG), an elective open heart procedure in hemodynamically stable patients taking their standard cardiac treatments and under the care of the cardiologist, and 11 required valve replacement. EAT samples were collected just from these 32 patients during surgery. Due to the lower amount of tissue isolated during surgery, 22 samples were used for microarray studies and 10 samples for protein expression studies. Blood samples for plasma quantification of PCSK9 were obtained from the total of patients and CTR subjects. Written informed consent was obtained from all participants. The study was approved by the local ethics committee (ASL Milano Due, protocol 2516) and conducted in accordance with the Declaration of Helsinki, as revised in 2013, and Good Clinical Practice guidelines.

### 2.2. Biochemical Parameters

Blood samples were collected after overnight fasting, into pyrogen-free EDTA tubes or in tubes for serum collection. EDTA plasma samples for nonroutine assays were obtained after centrifugation at 1200 g for 15 min and immediately stored at -20°C until subsequent analyses. A cobas 6000 analyzer and commercial kits (Roche Diagnostics, Milan, Italy) were used for the quantification of routine biochemical parameters, as previously reported [24–27]. LDL-cholesterol was calculated with the Friedewald formula. The homeostasis model assessment of insulin resistance (HOMA-IR) was calculated using the following equation: HOMA‐IR = (fasting insulin [*μ*U/mL] × fasting glucose [mmol/L])/22.5.

### 2.3. Anthropometric Measures

Weight, height, and waist circumference (WC) were directly measured at hospital admission. Weight and height were recorded to the nearest 0.1 kg and 0.5 cm using standard scales and stadiometers. WC was measured using a flexible tape. Body mass index (BMI) and waist-to-height ratio (WHtR) were then calculated as weight (kg)/height^2^ (m^2^) and WC (cm)/height (cm), respectively. As defined by WHO, patients were classified as normal weight (BMI 18.5-24.9 kg/m^2^), overweight (BMI 25.0-29.9 kg/m^2^), and obese (BMI ≥ 30.0 kg/m^2^).

### 2.4. EAT Thickness Measurement

All patients underwent standard echocardiography using commercially available equipment (Vingmed-System Five; General Electric, Horten, Norway). Briefly, EAT was identified as the echo-free space between the outer wall of the myocardium and the visceral layer of the pericardium. EAT thickness was measured perpendicularly on the free wall of the right ventricle at the end-systole in three cardiac cycles. The parasternal long-axis view allowed for the most accurate measurement of EAT on the right ventricle, with optimal cursor beam orientation in each view. Maximum EAT thickness was measured at the point on the free wall of the right ventricle along the midline of the ultrasound beam, perpendicular to the aortic annulus, used as the anatomical landmark for this view [28]. The average value of three cardiac cycles was calculated and used for analysis.

### 2.5. EAT Collection

Before starting cardiopulmonary bypass pumping, a sample of EAT adjacent to the proximal right coronary artery was harvested. For RNA and protein expression studies, EAT samples were stored in an Allprotect Tissue Reagent (QIAGEN, Hilden, Germany) at -20°C.

### 2.6. RNA Extraction and Microarray Analysis

Total RNA was extracted from tissue with the RNeasy Lipid Tissue Kit according to the manufacturer's procedure (QIAGEN). RNA concentration was quantified by NanoDrop 2000 (Thermo Scientific, Wilmington, Germany), and RNA integrity was assessed using the Agilent RNA 6000 Nano Kit and the Agilent 2100 Bioanalyzer (Agilent Technologies, Santa Clara, CA). Gene expression was analysed by a one-color microarray platform (Agilent): 50 ng of total RNA was labeled with Cy3 using the Agilent Low Input Quick Amp Labeling Kit-1 color, according to the manufacturer's directions. cRNA was purified with the RNeasy Mini Kit (QIAGEN), and the amount and labeling efficiency were measured with NanoDrop. For hybridization, we used an Agilent Gene Expression Hybridization Kit and scanned with the Agilent G2565CA Microarray Scanner System. Data were processed using Agilent Feature Extraction Software (10.7) with the single-color gene expression protocol, and raw data were analyzed with ChipInspector Software (Genomatix, Munich, Germany). In brief, raw data were normalized on the single-probe level based on the array mean intensities, and statistics were calculated based on the SAM algorithm by Tusher et al. [29]. Fold changes were calculated from normalized data.

### 2.7. PCKS9 Assays (ELISA)

Circulating levels of PCKS9 were measured on EDTA-plasma samples according to the manufacturer's directions with the PCSK9 Quantikine ELISA Kit (SPC900) from R&D Systems (Minneapolis, MN). The mean minimum detectable dose was 0.096 ng/mL [30]. The maximum intra- and interassay coefficients of variation were 6.5% and 5.9%, respectively.

### 2.8. Western Blot

EAT samples of heart tissues were homogenized with TissueLyser II (QIAGEN) in an ice-cold RIPA lysis buffer (50 mM Tris-HCl, pH 7.5, 150 mM NaCl, 1 mM EDTA, 1 mM EGTA, 1% Triton X-100, 0.1% SDS, 0.5% Na-deoxycholate, and 50 mM sodium fluoride) containing 1% protease inhibitor cocktail (Sigma-Aldrich, Milan, Italy). The homogenate was kept on ice for 30 min, centrifuged at 500 rpm for 10 min at 4°C, and the resulting supernatant centrifuged at 13,200 rpm for 15 min at 4°C. Protein concentration was determined with the Quantum Protein Assay Kit, based on a BCA reagent (EuroClone, Milan, Italy). Equal amounts of protein samples (30 *μ*g) were suspended in a Laemmli sample buffer and separated using 4-20% Mini-PROTEAN TGX Stain-Free Gels and a Tris/Glycine/SDS running buffer (Bio-Rad). The separated proteins were then transferred from the gel to a nitrocellulose membrane using the Trans-Blot Turbo Mini Nitrocellulose Transfer Packs and the Trans-Blot Transfer System (Bio-Rad). The membranes were blocked with 5% dry milk in Tris-buffered saline/0.1% Tween 20 for 1 h at room temperature, and the blots were then incubated overnight at 4°C with a diluted solution of the primary anti-PCSK9 antibody (1 : 500) (GeneTex, Alton Pkwy, Irvine, CA, USA). The subsequent incubation with a secondary antibody conjugated with peroxidase (1 : 5000) was performed at room temperature for 2 h. Immunoreactivity was detected by a working solution (Clarity Western ECL Substrate, Bio-Rad) and the ChemiDoc Touch System.

### 2.9. Statistical Analysis

Quantitative variables are expressed as median and 25th-75th percentiles or mean ± SD. Qualitative variables are summarized as numbers and percentages. The normality of data distribution was assessed with the Kolmogorov-Smirnoff test. Comparison between two groups was performed by *t*-test or Mann-Whitney tests for continuous variables. Fisher's exact test was used for nominal variables. Relations between parameters were examined with the Spearman correlation test. Data were analyzed using the GraphPad Prism 5.0 biochemical statistical package (GraphPad Software, San Diego, CA). A *p* value < 0.05 was considered significant.

## 3. Results

### 3.1. Patient Characteristics

The main demographic, anthropometric, clinical, and biochemical characteristics of patients are shown in Table 1. Mean age (± standard deviation, SD) was 65.99 ± 10.64 years and males were the majority (88.24%). According to the body mass index (BMI), roughly 50% were overweight and 30% obese. EAT thickness ranged from 3 to 12 mm (mean value ± SD: 7.40 ± 2.55 mm). Relative to lipid profile, this was in the normal range: total cholesterol 148.00 ± 27.74 mg/dL, LDL-cholesterol (LDL-C) 79.96 ± 24.74 mg/dL, and HDL-C 47.07 ± 26.65 mg/dL. Sixty-seven % (67.65%) were under statin treatment. Out of 68 patients, 48 had a diagnosis of coronary artery disease (CAD) and 11 of valve disease. Thirty-two underwent open heart surgery for coronary bypass grafting (CABG, *n* = 21) or valvular replacement (VR, *n* = 11). As shown in Table 1, patients in this subgroup had a lower incidence of diabetes with lower levels of HbA1c.

### 3.2. PCSK9 Expression in EAT

**Figure 1:**
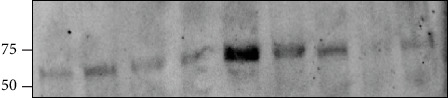
PCSK9 expression in Epicardial adipose tissue (EAT). Western blot analysis shows PCSK9 expression in EAT samples.

PCSK9 expression in EAT was evaluated at both the gene and protein levels. PCSK9 gene expression was evaluated by microarray analysis (mean expression level ± SD: 6.14 ± 4.57 arbitrary unit), with the PCSK9 local level being positively correlated with EAT thickness (*r* = 0.502, *p* = 0.017). Since statins increase the PCSK9 levels, PCSK9 gene expression was evaluated in both patients taking and patients not taking statins: no difference has been observed between the two groups (*p* > 0.05) (data not shown). The immunoreactive band of 62-66 kDa confirmed the protein expression of PCSK9 (Figure 1).

### 3.3. Correlation of PCSK9 Expression with Genes Involved in the Inflammatory Response in EAT

The expression of 142 genes (supplementary Table 1) involved in the inflammatory response in EAT was analyzed and correlated with the PCSK9 levels. Twenty-two genes were found to be directly correlated with PCSK9, especially those encoding for chemokines and chemokine receptors, which exert chemotactic activity for lymphocytes, monocytes, and granulocytes, and interleukins. Names and functions of the genes which correlate with PCSK9 are reported in Table 2.

### 3.4. Plasma Levels of PCSK9

**Figure 2:**
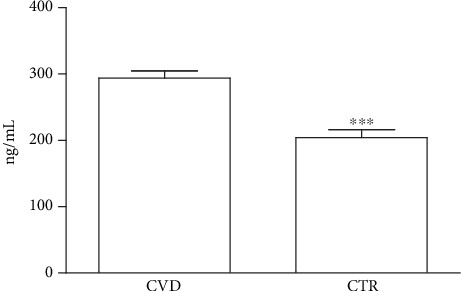
Quantification of plasma PCSK9 levels in cardiovascular disease patients (CVD) and healthy individuals (CTR). PCSK9 levels were higher in CVD patients than CTR. Data are expressed as mean ± SD. ^∗∗∗^*p* < 0.001 vs. CTR.

Plasma levels of PCSK9 were measured in CVD patients and in a group of 29 CTR. PCSK9 was higher in CVD patients than in CTR (293.90 ± 91.13 vs. 204.20 ± 64.61 ng/mL, respectively; *p* < 0.0001) (Figure 2). Among the CVD patients, circulating PCSK9 levels were higher in those given statins (246.10 ± 84.72 ng/mL vs. 309.20 ± 85.95 ng/mL; *p* < 0.01). EAT thickness was also higher in CVD patients than in CTR (7.40 ± 2.55 mm vs. 3.79 ± 1.59 ng/mL; *p* < 0.0001). Correlation analyses of the plasma PCSK9 levels with PCSK9 expression in EAT and EAT thickness were not statistically significant (*p* > 0.05).

## 4. Discussion

In the present study, for the firt time, we have demonstrated that EAT is a source of PCSK9 and that EAT inflammation is associated with local PCSK9 expression, regardless of the circulating PCSK9 levels.

The liaison between PCSK9 and adipose tissue still lacks evidence. It was previously demonstrated that PCSK9 is expressed and secreted by viscaral adipose tissue (VAT) and correlated with BMI [31]. PCSK9 seems to reduce VAT accumulation independently of the LDLR by promoting the degradation of the very-low density lipoprotein receptor (VLDLR) in adipocytes, thereby limiting fatty acid internalization [32, 33]. *PCSK9 R46L* carriers, compared with noncarriers, have shown increased BMI, a rise in the percentage of total and android fat masses, and a higher EAT thickness. These observations were replicated by the same group in PCSK9 KO mice, that showed an increment in visceral adipose tissue, but not in the subcutaneous ones [32, 33]. Conversely, we observed positive correlations of PCSK9 expression in EAT with EAT amount and local inflammation, but not with the PCSK9 circulating levels. Overall, inflammation may be the main link between EAT enlargement and PCSK9 expression. Indeed, our and other groups found that in CVD patients, EAT is infiltrated by macrophages polarized toward a proinflammatory M1 state and T- and B-lymphocytes and that the local inflammatory status is strongly associated with EAT adipocyte size, EAT amount, and lipid droplet degeneration [21, 34, 35]. Therefore, we can hypothesize that EAT inflammation may upregulate PCSK9 expression which in turn can exert proinflammatory and detrimental effects locally and on tissues lying in contiguity with EAT, such as the myocardium and coronary vessels. The bidirectional association between inflammation and PCSK9 has been previously demonstrated both *in vitro* and in the animal models [4, 36]. PCSK9 silencing in macrophages can partially reduce oxLDL-induced-cytokine production, with PCSK9 KO mice showing a reduced inflammatory response [37]. PCSK9 stimulates the expression of proinflammatory cytokines after exposure to LPS, and PCSK9 downregulation in atherosclerotic plaques reduces macrophage infiltration and cytokine secretion [7, 37–39]. Although our study did not explore the mechanisms linking inflammation to PCSK9 expression, it confirmed the existence of correlations also in EAT. Looking at the molecules associated with PCSK9 expression, it can be noted that they are mainly chemokines, cytokines, and other molecules involved in chemotaxis, differentiation, and activation of different leukocyte populations as well as proinflammatory mediators that can amplify local inflammation through NF-*κ*B activation. In particular, the association previously observed *in vitro* between PCSK9 and CXCL2 [7] has been detected in EAT too, thus confirming that PCSK9 can play a role in monocyte recruitment.

The protein expression of PCSK9 in EAT argues that PCSK9 is part of the EAT secretome. Therefore, in addition to increasing local EAT inflammation, PCSK9 derived from EAT may directly affect the coronary vessels and the myocardium, thus increasing CV risk. This amount of EAT-secreted PCSK9 is added to the amount that can be derived from the circulation. The finding that CVD patients have higher plasma PCSK9 levels is in line with previous reports showing PCSK9 to be associated with the Framingham Risk Score [40] or with inflammation in the acute phase and hypercholesterolemia in patients with acute coronary syndrome [41]. In our study, we did not find any association of plasma PCSK9 with local EAT expression and inflammation. Therefore, EAT-derived PCSK9 may play an additional and independent risk to CVD. Recently, it has also been demonstrated that PCSK9 is not only implicated in atherogenesis but can also determine the development of infarct size and affect contractive function of cardiomyocytes [5, 42].

From a clinical point of view, in atherosclerotic plaques, PCSK9 monoclonal antibodies can reduce macrophage content through the reduction of CCR2 expression in monocytes with a consequent impairment in their migratory capacity [43, 44]. Whether this treatment may affect EAT inflammation, its metabolism, and amount is unknown; therefore, additional studies are necessary to demonstrate any potential effect of reduced PCSK9 levels on EAT and EAT-associated CV risk.

It is important to point out that about 50% of our patients were under statin therapy. Statin use is associated with a reduction in EAT thickness and inflammation, supporting the hypothesis of a direct action of statins on the EAT secretory profile [45, 46]. Targeting some inflammatory mediators, such as IL1*β*, reduces CV events, regardless of LDL-C lowering [47]. Differently, statins may upregulate the expression of PCSK9 via SREBP-2 [48]. Therefore, the PCSK9 levels in EAT may be influenced by the direct effect of statins on its expression and the modulation of local inflammation too. Notably, no rise in PCSK9 protein expression was found in EAT from patients taking statins. However, no data are available relative to the association between modulation of EAT inflammation and PCSK9 levels.

Our study has some limitations. First, the amount of EAT collected during surgery did not allow us to evaluate EAT transcriptomes and proteomes on the same samples. Second, we did not have a surgical control group used to compare PCSK9 and the other genes analyzed. Third, our study group is composed of both CABG and VR patients. Although the mechanisms driving the two pathologies are different, many studies demonstrated the involvement of EAT amount and secretome in the progression of both diseases [14, 49–52]. Fourth, since our study is cross-sectional, our findings cannot imply causality. In conclusion, we provided evidence for the possible role of PCSK9 as an EAT-derived risk factor for CVD. In the clinical setting, reducing both the EAT inflammation and the PCSK9 local levels might have beneficial effect on EAT metabolism and EAT-related CV risk, but this needs further investigations.

## Figures and Tables

**Table 1:** Demographic, anthropometric, clinical, and biochemical characteristics.

	CVD (*n* = 68)	CAGB+VR (*n* = 32)
Age (years)	65.99 ± 10.64, 66.00 (59.00-74.75)	66.31 ± 10.96, 68.50 (55.75-77.00)
Male gender (*n*, %)	60, 88.24%	28, 87.50%
BMI	28.50 ± 4.69, 27.80 (25.40-30.39)	28.07 ± 4.95, 26.00 (24.63-30.57)
WC (cm)	106.00 ± 13.97, 105.00 (102.00-116.00)	104.90 ± 12.28, 104.00 (102.00-110.80)
EAT thickness (mm)	7.40 ± 2.55, 8.00 (5.00-9.00)	6.70 ± 2.27, 7.00 (5.00-8.00)
Fasting glucose (mg/dL)	102.70 ± 40.22, 93.00 (81.75-107.00)	93.58 ± 15.76, 94.00 (79.00-103.00)
Fasting insulin (microU/mL)	9.51 ± 6.66, 8.15 (5.87-11.39)	9.24 ± 4.19, 8.68 (6.27-12.22)
HbA1c (%)	5.49 ± 1.33, 5.41 (4.93-6.04)	4.90 ± 1.09, 5.17 (4.06-5.76)^∗^
HOMA-IR	2.21 ± 1.88, 1.57 (1.12-2.71)	1.64 ± 0.73, 1.61 (1.02-2.23)
Total cholesterol (mg/dL)	157.20 ± 36.33, 154.50 (134.50-179.80)	161.80 ± 37.24, 159.50 (132.80-182.80)
LDL-cholesterol (mg/dL)	89.22 ± 32.39, 83.80 (68.20-108.70)	93.56 ± 35.06, 92.20 (65.20-115.00)
HDL-cholesterol (mg/dL)	54.54 ± 37.60, 43.00 (33.50-53.00)	70.97 ± 48.22, 48.00 (40.00-103.00)
Triglycerides (mg/dL)	119.90 ± 68.68, 105.50 (74.00-152.50)	98.58 ± 56.11, 97.00 (43.00-122.00)
CRP (mg/dL)	0.99 ± 1.92, 0.30 (0.10-0.80)	0.71 ± 1.32, 0.20 (0.10-0.50)
ALT (U/L)	34.66 ± 35.79, 25.00 (15.00-39.00)	33.74 ± 42.11, 24.00 (15.00-32.00)
AST (U/L)	30.82 ± 31.93, 21.00 (16.00-39.00)	35.35 ± 41.39, 21.00 (16.00-31.00)
Smoking (*n*, %)	38, 55.88%	15, 46.88%
Hypertension (*n*, %)	53, 77.94%	25, 78.13%
History of cardiovascular diseases (*n*, %)	25, 36.76%	11, 34.38%
Dyslipidemia (*n*, %)	46, 67.65%	18, 56.25%
Diabetes (*n*, %)	17, 25.00%	2, 6.25%∗
Aspirin (*n*, %)	48, 70.59%	20, 62.50%
ACEI/ARB (*n*, %)	46, 67.64%	23, 71.88%
Antidiabetic agents	15, 22.07%	2, 6.25%
*β*-Blockers (*n*, %)	39, 57.35%	16, 50.00%
Calcium channel blockers (*n*, %)	15, 22.06%	6, 18.75%
Statins (*n*, %)	46, 67.65%	18, 56.25%

The table shows the main characteristics of the whole studied population (CVD: cardiovascular disease patients) and the subgroup of patients who underwent either CABG (coronary artery bypass grafting) or VR (valvular replacement). Data are expressed as mean ± standard deviation, median (25th-75th percentiles) or number and proportions. ACEI: angiotensinogen-converting enzyme inhibitor; ALT: alanine aminotransferase; AST: aspartate aminotransferase; ARB: angiotensin receptor blockade; BMI: body mass index; CRP: C reactive protein; EAT: epicardial adipose tissue; HbA1c: glycated hemoglobin; HOMA-IR: homeostatic model assessment of insulin resistance; WC: waist circumference. ^∗^*p* < 0.05.

**Table 2:** Correlation analyses of PCSK9 with genes involved in the inflammatory response in EAT.

Genes	Family group	Function	Correlation coefficient, *p* value
C5	Regulation of inflammation	Member of the complement system: mediator of local inflammatory process	0.446, 0.038
CCL11	Chemokine	Chemotactic activity for eosinophils	0.508, 0.016
CCL20	Chemokine	Chemotactic activity for lymphocytes	0.470, 0.028
CCL21	Chemokine	Chemotactic for thymocytes and activated T-cells, but not for B-cells, macrophages, or neutrophils	-0.447, 0.037
CCR6	Chemokine receptor	Ligand for macrophage inflammatory protein 3 alpha	0.416, 0.050
CEBPB	Regulation of inflammation	Transcription factor regulating the expression of genes involved in immune and inflammatory responses	0.433, 0.044
CSF3	Regulation of inflammation	It control of production, differentiation, and function of granulocytes	0.423, 0.049
CXCL2	Regulation of inflammation	Produced by activated monocytes and neutrophils and expressed at sites of inflammation and may suppress progenitor cell proliferation	0.459, 0.032
CXCL6	Regulation of inflammation	Chemotactic for neutrophil granulocytes	0.459, 0.031
CXCL10	Regulation of inflammation	Proinflammatory cytokine that is involved in a wide variety of processes such as chemotaxis, differentiation, and activation of peripheral immune cells, regulation of cell growth, apoptosis, and modulation of angiostatic effects	0.456, 0.033
CXCL13	Regulation of inflammation	Chemotactic for B-lymphocytes	0.591, 0.004
CXCR5	Regulation of inflammation	Binds to B-lymphocyte chemoattractant (BLC) and is involved in B-cell migration	0.431, 0.045
FASLG	Other molecules	Immune system regulation, including activation-induced cell death of T-cells and cytotoxic T lymphocyte-induced cell death	0.634, 0.002
IFN*γ*	Other molecules	Potent activator of macrophages	0.443, 0.039
IL1*β*	Interleukins	Induces prostaglandin synthesis, neutrophil influx and activation, T-cell activation and cytokine production, and B-cell activation	0.445, 0.038
IL10	Interleukins	Major immune regulatory cytokine that acts on many cells of the immune system where it has profound anti-inflammatory functions, limiting excessive tissue disruption caused by inflammation	0.421, 0.050
IL17A	Interleukins	Proinflammatory cytokine produced by activated T-cells	0.476, 0.025
IL17F	Interleukins	Expressed by activated T-cells and has been shown to stimulate the production of several other cytokines, including IL6, IL8, and CSF2/GM_CSF	0.499, 0.018
IL33	Interleukins	Acts as a chemoattractant for Th2 cells and may function as an “alarmin,” that amplifies immune responses during tissue injury	-0.429, 0.046
KNG1	Other molecules	Mediator of inflammation	0.439, 0.041
RIPK4	Regulation of inflammation	Activator of NF-kappa-B	0.572, 0.005
TLR9	Regulation of inflammation	TLR9 is a nucleotide-sensing TLR which is activated by unmethylated cytidine-phosphate-guanosine (CpG) dinucleotides. Acts via MYD88 and TRAF6, leading to NF-kappa-B activation, cytokine secretion, and the inflammatory response	0.431, 0.046
TLR10	Regulation of inflammation	Acts via MYD88 and TRAF6, leading to NF-kappa-B activation, cytokine secretion, and the inflammatory response	0.484, 0.027
TNFSF9	Other molecules	Induces the proliferation of activated peripheral blood T-cells	0.435, 0.043

The table reports correlations of PCSK9 level with genes involved in the inflammatory response in EAT. Spearman's correlation coefficients and corresponding *p* values are reported.

## Data Availability

The data used to support the findings of this study are available from the corresponding author upon request.

## Supplementary Materials

Supplementary Materials Supplementary Table 1 describes the list of genes that have been analyzed in the study and correlated with PCSK9 expression in EAT.
