# Supplementary material for: PCSK9 Expression in Epicardial Adipose Tissue: Molecular Association with Local Tissue Inflammation
Source: Mediators Inflamm. 2020 Jun 4;2020:1348913. doi: 10.1155/2020/1348913 (PMC7292972; doi:10.1155/2020/1348913)
Supplement: Supplementary Materials — Supplementary Table 1 describes the list of genes that have been analyzed in the study and correlated with PCSK9 expression in EAT. [file 1348913.f1.docx]

**List of the gene involved in the inflammatory response evaluated in EAT**

AIMP1 BCL6 BMP2 C3 C3AR1 C5 CCL1 CCL11 CCL13 CCL15 CCL16 CCL17 CCL19 CCL2 CCL20 CCL21 CCL22 CCL23 CCL24 CCL26 CCL3 CCL4 CCL5 CCL7 CCL8 CCR1 CCR2 CCR3 CCR4 CCR5 CCR6 CCR7 CCR8 CD14 CD40 CD40LG CEBPA CEBPB CEBPD CEBPE CRP CSF1 CSF2 CSF3 CX3CL1 CX3CR1 CXCL1 CXCL10 CXCL11 CXCL12 CXCL13 CXCL2 CXCL3 CXCL5 CXCL9 CXCR1 CXCR2 CXCR4 CXCR5 CXCR6 CXCR7 FASLG FOS IFNA2 IFNG IL10 IL10RA IL10RB IL13 IL15 IL16 IL17A IL17B IL17C IL17D IL17F IL18 IL1A IL1B IL1R1 IL1RAP IL1RN IL21 IL22 IL23A IL23R IL27 IL3 IL33 IL5 IL5RA IL6 IL6R IL7 IL9 IL9R ITGB2 KNG1 LY96 MIF MYD88 NAMPT NFKB1 NOS2 NR3C1 NR3C2 OSM PTGS1 PTGS2 RIPK1 RIPK2 RIPK3 RIPK4 SELE SPP1 TIRAP TLR1 TLR10 TLR2 TLR3 TLR4 TLR5 TLR6 TLR7 TLR8 TLR9 TNF TNFSF10 TNFSF11 TNFSF12 TNFSF13 TNFSF13B TNFSF14 TNFSF15 TNFSF18 TNFSF4 TNFSF8 TNFSF9 TOLLIP VEGFA VEGFB VEGFC
